# High Pressure Freezing/Freeze Substitution Fixation Improves the Ultrastructural Assessment of *Wolbachia* Endosymbiont – Filarial Nematode Host Interaction

**DOI:** 10.1371/journal.pone.0086383

**Published:** 2014-01-17

**Authors:** Kerstin Fischer, Wandy L. Beatty, Gary J. Weil, Peter U. Fischer

**Affiliations:** 1 Infectious Diseases Division, Department of Internal Medicine, Washington University School of Medicine, St. Louis, Missouri, United States of America; 2 Imaging Facility, Department of Molecular Microbiology, Washington University School of Medicine, St. Louis, Missouri, United States of America; University of Poitiers, France

## Abstract

**Background:**

*Wolbachia* α-proteobacteria are essential for growth, reproduction and survival for many filarial nematode parasites of medical and veterinary importance. Endobacteria were discovered in filarial parasites by transmission electron microscopy in the 1970’s using chemically fixed specimens. Despite improvements of fixation and electron microscopy techniques during the last decades, methods to study the *Wolbachia*/filaria interaction on the ultrastructural level remained unchanged and the mechanisms for exchange of materials and for motility of endobacteria are not known.

**Methodology/Principal Finding:**

We used high pressure freezing/freeze substitution to improve fixation of *Brugia malayi* and its endosymbiont, and this led to improved visualization of different morphological forms of *Wolbachia*. The three concentric, bilayer membranes that surround the endobacterial cytoplasm were well preserved. Vesicles with identical membrane structures were identified close to the endobacteria, and multiple bacteria were sometimes enclosed within a single outer membrane. Immunogold electron microscopy using a monoclonal antibody directed against *Wolbachia* surface protein-1 labeled the membranes that enclose *Wolbachia* and *Wolbachia-*associated vesicles. High densities of *Wolbachia* were observed in the lateral chords of L4 larvae, immature, and mature adult worms. Extracellular *Wolbachia* were sometimes present in the pseudocoelomic cavity near the developing female reproductive organs. *Wolbachia*-associated actin tails were not observed. *Wolbachia* motility may be explained by their residence within vacuoles, as they may co-opt the host cell’s secretory pathway to move within and between cells.

**Conclusions/Significance:**

High pressure freezing/freeze substitution significantly improved the preservation of filarial tissues for electron microscopy to reveal membranes and sub cellular structures that could be crucial for exchange of materials between *Wolbachia* and its host.

## Background

Most medically and economically important filarial parasites in the subfamilies *Onchocercinae* and *Dirofilariinae* depend on *Wolbachia* endosymbionts for development, reproduction and survival. Intracellular bacteria were first observed in filarial worms in early ultrastructural studies of *Dirofilaria immitis*, *Brugia pahangi*, *Brugia malayi* and *Onchocerca volvulus* [1]–[3]. Subsequent studies provided additional morphological information on these bacteria [4]–[7]. However, it was not until the late 1990’s that these endobacteria were shown to belong to the genus *Wolbachia*, and this led to research that explored the bacteria as a novel drug target for filariasis [8]–[11].

Morphological studies by our group on the dynamics of *Wolbachia* distribution during the life cycle of *B. malayi* showed that *Wolbachia* densities are relatively low in microfilariae and vector stage larvae. Bacterial densities increase exponentially in the lateral chords of developing worms within the vertebrate host, and they later invade the growing ovaries of immature female worms [12]. Other authors have confirmed this infection process across tissue membranes. It now appears well established that *Wolbachia* are not only passively transferred to daughter cells in the course of cell division, but they also are able to cross cell membranes [13]–[15]. Ultrastructural studies with improved morphological preservation of fine structure of the bacteria and nematode tissues may provide new insight regarding *Wolbachia* movement and bacteria-host cell interactions. Initial ultrastructural studies of *Wolbachia* in filarial nematodes described endobacterial morphology extensively and reported that the bacterial cytoplasm was surrounded by three membranes [1], [2]. Subsequent examinations of filarial parasites show varying levels of morphological quality, but studies of *Wolbachia* ultrastructure were often hampered by suboptimal preservation of membranes and organelles [12], [16]–[19].

Despite technical advances in electron microscopy in the last two decades, protocols for detecting *Wolbachia* and their interaction with filarial host tissues have changed very little since the bacteria were discovered in the 1970’s. High pressure freezing/freeze substitution fixation (HPF/FS) is a relatively new fixation method for transmission electron microscopy (TEM) that is especially useful for studies of cell and organelle membranes [20]. Comparison of chemical fixation and HPF/FS in platyhelminthes demonstrated the superiority of HPF/FS for the detection of membranes and organelles such as the ciliary rootlet system [21]. This fixation method also preserves antigenic properties of proteins, making it an excellent choice for immunogold electron microscopy [22].

Therefore, the objective of the present study was to use HPF/FS for TEM and immunogold TEM to better assess the relationship of *Wolbachia* to membranes and host tissues. This revealed many new and exciting features and suggested new hypotheses regarding *Wolbachia*-filaria interactions.

## Results and Discussion

### Comparison of Chemical and HPF/FS Fixation

**Figure 1:**
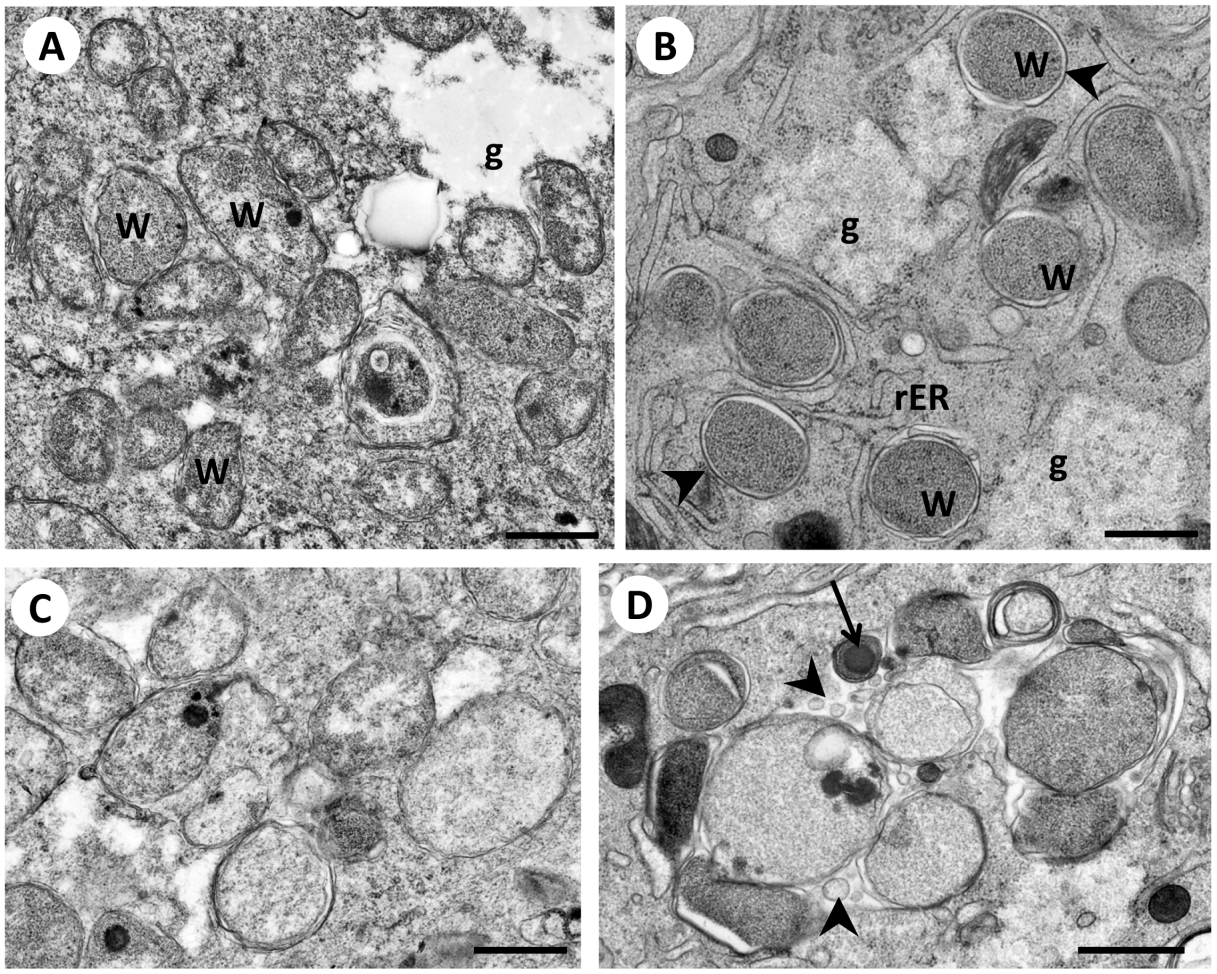
Comparison of chemical fixation and HPF/FS fixation of 5 week old female *B. malayi*. **Panel A** *Wolbachia* in the lateral chord of a chemically fixed sample. **B** The same region as in A in a specimen fixed by HPF/FS. Note the improved preservation of membranes (arrowheads) and subcellular structures. **C** Cluster of *Wolbachia* within the lateral chord of a chemically fixed specimen. **D** Similar cluster as in C in a HPF/FS fixed specimen. Note the pleomorphy of the endobacteria (e.g. small electron dense bacteria with large nucleoid, arrow, next to large bacteria) and the small vesicles in the same area (arrowheads). W, *Wolbachia*; rER, rough endoplasmic reticulum; g, glycogen. Scale bar corresponds to 500 nm.

Ultrastructural studies on various single cell as well as on multicellular organisms indicate that HPF/FS is superior to conventional, chemical fixation for preservation of membranes and subcellular structures [20], [21]. For example, studies using HPF/FS improved the understanding of the ultrastructure and functional anatomy of the model nematode *Caenorhabditis elegans* [22], [23]. Therefore we postulated that HPF/FS would improve the preservation of filarial nematodes and their endosymbiont for ultrastructural analysis. While membranes can be detected using chemical fixation and Epon embedding, their relation to *Wolbachia* is often difficult to recognize (Fig. 1A, C). Glycogen, which is often seen in the vicinity of *Wolbachia* in the lateral chord can be easily differentiated by HPF/FS, but is difficult to recognize in chemically fixed specimens (Fig. 1A, B). Undoubtedly using aldehydes as fixatives resulted in good morphological preservation in former studies [24], [25]. However, it is known that LR White embedded material results in “halo” artifacts surrounding *Wolbachia* and it can be a problem to distinguishing *Wolbachia* from the host cytoplasm in Epon embedded material [26]. With HPF/FS fixed specimens none of these problems were encountered.

### Morphology of *Wolbachia*

**Figure 2:**
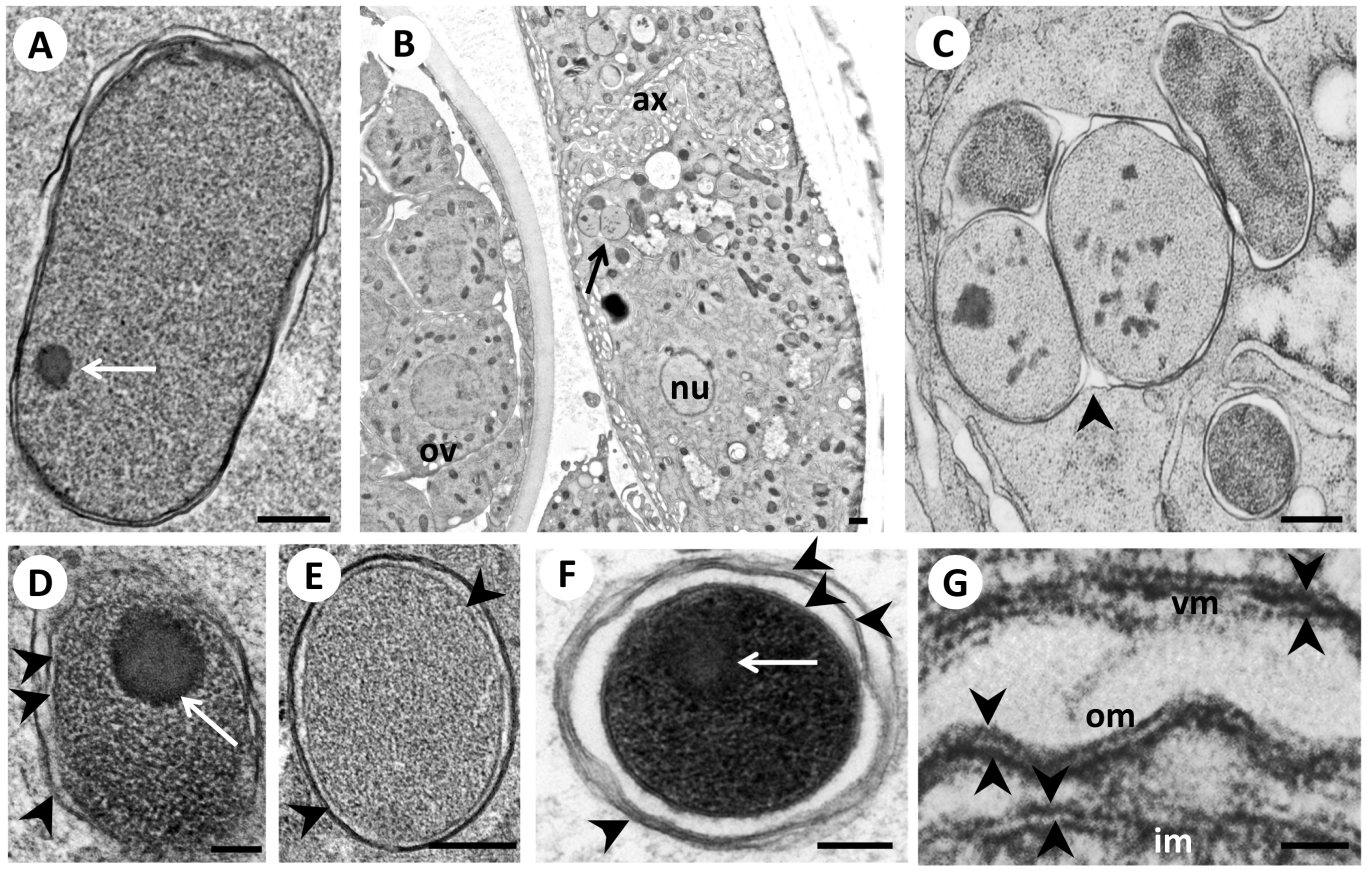
Pleomorphy of *Wolbachia* in female *B. malayi*. **Panel A** Large bacillary *Wolbachia* in the lateral chord of a 3 week old fourth stage larva. The arrow points to a small nucleoid. **B** Cross-section of the midbody region of a 5 week old immature female. A large vacuole containing a cluster of *Wolbachia* (arrow) is seen at the inner part of the lateral chord. **C** shows a higher power view of B with ‘inclusion-like’ vacuole containing three *Wolbachia*. Note the varying density of the cytoplasm in the bacteria. The arrow head indicates the common vacuole membrane. **D** *Wolbachia* with a large and prominent nucleoid (arrow) in the lateral chord of a 6 week old immature female. **E** Large coccoid *Wolbachia* in the lateral chord of a 12 week old (mature) female. **F** Coccoid, electron dense *Wolbachia* in morula stage embryo within the uterus of a 12 week old female. Note the large nucleoid (arrow) and the expanded space between the membranes. Arrowheads indicate membranes. **G** The three membranes surrounding *Wolbachia* in the morula stage embryo within a 12 week old female. Note the symmetrical inner and outer leaflets (arrowheads) of the cytoplasmic inner membrane (im), the outer membrane (om) and the vacuole membrane (vm). Ov, ovary; nu, nucleus; ax, axons. Scale bar corresponds to 100 nm (A–F) or 25 nm (G).

*Wolbachia* endobacteria were highly pleomorphic in all developmental stages of the parasite that we studied (Fig. 2A–F, Table 1). *Wolbachia* distribution and morphology are stage and gender dependent, and vary by location in the worm (tissue and body region). For this study we decided to focus on developing females, because we wanted to evaluate *Wolbachia* morphology in embryonic stages. Pleomorphy was observed with regard to size, shape, and electron density of the cytoplasm. We observed striking variability in the appearance of the nucleoid, vesicle formation, the number of membranes surrounding the bacteria, the space between membranes, vacuole morphology, and in patterns of *Wolbachia* aggregation. Relatively small (250–500 µm), electron dense, coccoid *Wolbachia* with thick membranes were typically seen in developing embryos in the uterus of 8–12 weeks old females (Fig. 2 F). Similar but less electron dense endobacteria were present in the lateral chords of 5–6 week old, immature females (Fig. 2D). In areas with high *Wolbachia* densities, clustered *Wolbachia* sometimes were present in a single vacuole (Fig. 2B). Interestingly, *Wolbachia* within a vacuole were not always morphologically identical, and this suggests that the cells may be in different stages of the cell cycle, perhaps due to asynchronous cell division within vacuoles.

**Table 1:** Ultrastructural characteristics of predominant *Wolbachia* forms of *B. malayi* found in the examined parasite material.

Stage examined	Localization of *Wolbachia*	Size range(nm)	Description	Remarks
8 to 12 week oldfemales	Developing embryos	250–500	Coccoid, electron dense, frequently with a darkernucleoid, 3 clear membranes, sometimes associatedwith small vesicles, single or in pairs	Oocytes contain less electron dense *Wolbachia*, singly or in clusters of 2–5
12 week old females	Lateral chords/hypodermis	400–800	Bacillary forms, less electron dense, often clustered,some groups in a large ‘inclusion-like’ vacuole, varyingin size and density, sometimes dividing, smallerforms with nucleoid, often in the vicinity of ribosomes,Golgi or glycogen	
8 week old females	Lateral chord/hypodermis	400–800	Similar to 12 week old females, but frequently associatedwith small and large vesicles	
2–4 week oldfemale/male L4	Lateral chords	250–900	Intermediate forms, highly pleomorphic, sometimessmall and electron dense, sometimes more greyish,often associated with vesicles. Multiple bacteria aresometimes present in one large ‘inclusion-like’ vacuole	
3–6 week oldL4/adults	Extracellular in the pseudocoelom	250–400	Small forms with no outer vacuole membrane,only single, non-dividing bacteria	Pseudcoelomic *Wolbachia* restricted to the vicinity of the (short) ovaries
5–6 week oldfemales	Lateral chords/hypodermis	250–900	Similar to *Wolbachia* in L4, higher total numberof bacteria	Few *Wolbachia* detected in the ovary; no embryos present in these immature worms

An early ultrastructural study described different morphological forms of endobacteria in *B. malayi* [1]. The author suggested that the endobacteria reside in vacuoles formed by the host’s membranes and that they are randomly distributed throughout the length of the lateral chord. While our results clearly show that intracellular *Wolbachia* are surrounded by three bilayer membranes and that the space between the membranes varies in different *Wolbachia* forms (compare Fig. 2E and 2F), they do not support Kozek’s suggestion that they are randomly distributed. Indeed, *Wolbachia* exhibit different distribution patterns in the lateral chords during different stages of worm development [12].

Lipopolysaccharides (LPS) present on the outer leaflets of the outer membrane of Gram negative bacteria are stained by heavy metals, and this can be observed by transmission electron microscopy. Previous studies showed that HPF/FS fixation is especially suitable for preserving this asymmetric bilayer membrane structure [27]. Although *Wolbachia* do not possess LPS [28], our results show strong staining of the outer and the inner leaflets of the bilayer membrane structure (Fig. 2G). This symmetric ultrastructural staining pattern is similar to the one recently reported for the rickettsial endosymbiont ‘MIDORIKO’ of green algae using HPF/FS fixation [29]. We did not observe host-derived actin tails in *Wolbachia* that are involved in intracellular mobility in some other α-proteobacteria such as *Rickettsia rickettsii* [30], [31]. For *Drosophila* it was reported that *Wolbachia* localization is dependent on microtubules, but not on the actin-based cytoskeleton [32].The absence of actin tails in *Wolbachia* of *B. malayi* suggests that this also applies to filarial *Wolbachia*.

### *Wolbachia* Form and Secrete Vesicles

**Figure 3:**
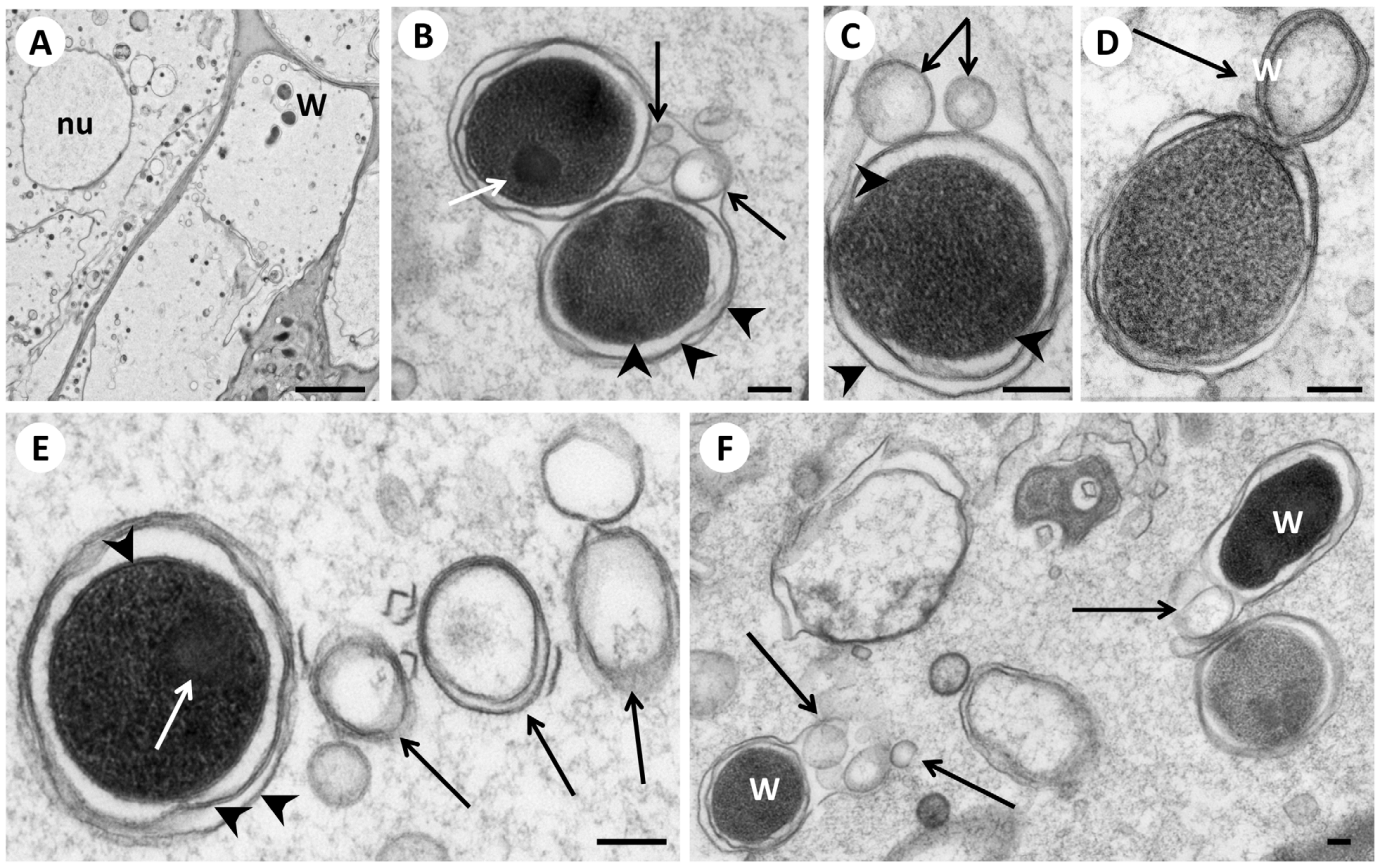
Formation and release of vesicles by *Wolbachia* in morula stage embryos in the uterus of a 12 week old female *B. malayi*. **Panel A** Overview of morula cells showing three electron dense *Wolbachia.* **B** Two *Wolbachia* surrounded by a common vacuole membrane. Note the nucleoid (white arrow) and the vesicles within the vacuole (black arrow). **C** Higher powered view of panel A with a coccoid *Wolbachia* with 2 large vesicles (arrows) within the same vacuole membrane. **D** *Wolbachia* with an attached large vesicle (arrow) with a double membrane. **E** *Wolbachia* next to several large vesicles (black arrow). Note the nucleoid (white arrow). **F** Overview of several *Wolbachia* (W) and *Wolbachia*-derived vesicles (arrows). Note the variable electron density of *Wolbachia.* Arrowheads indicate membranes. W, *Wolbachia*; nu, nucleus. Scale bar corresponds to 2 µm (**A**) or 100 nm (**B–F**).

**Figure 4:**
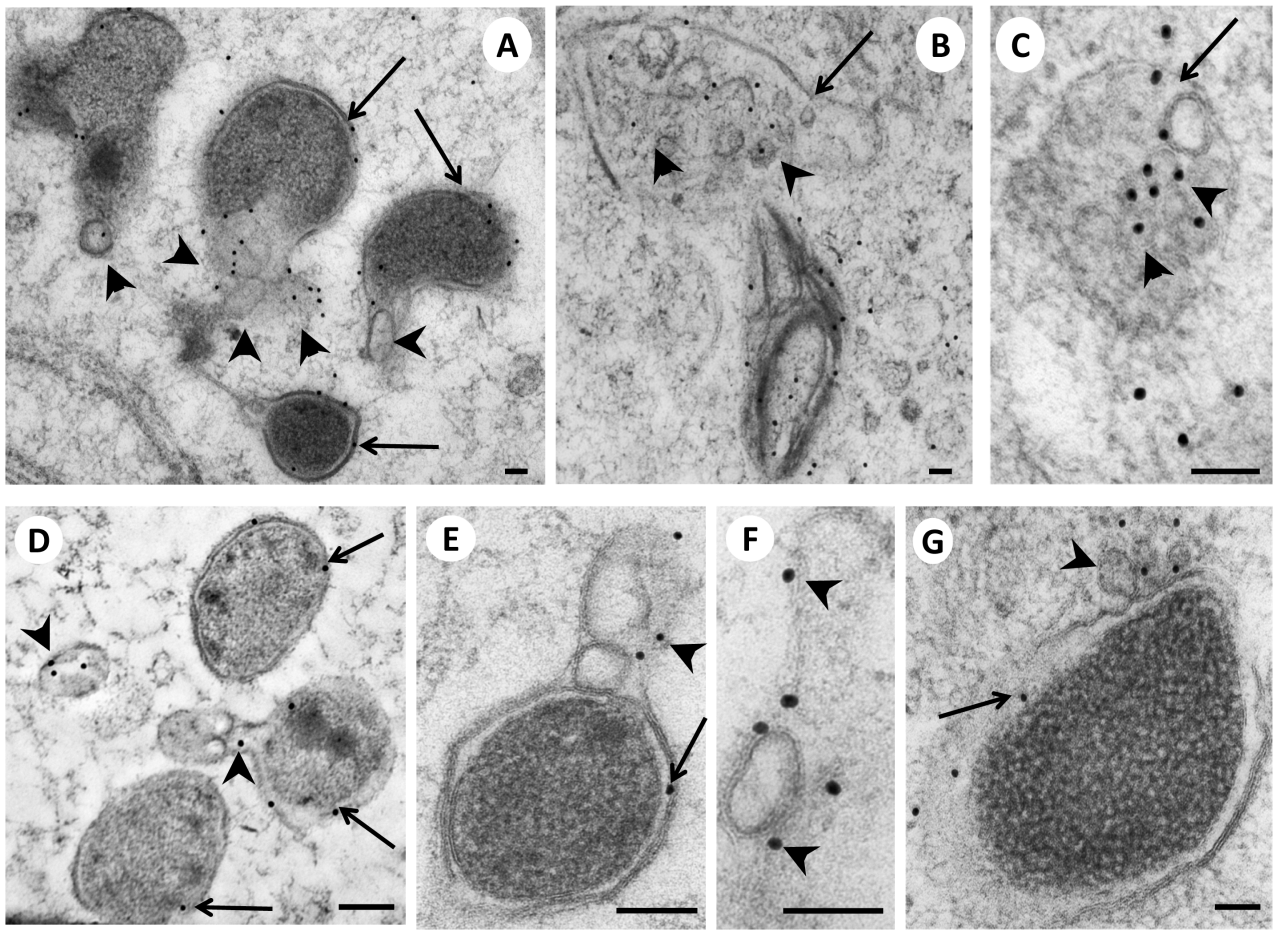
Immunogold-labeling of *Wolbachia* and their vesicles in the lateral chord and embryos of a 12 weeks old female *B. malayi* using mab WSP-1. **Panel A** Labeled *Wolbachia* (arrows) next to multiple labeled membrane vesicles (arrowheads) in an embryo. **B** Large vesicle (arrow) with an accumulation of many small labeled vesicles (arrowheads). **C** Higher powered view of a vesicle-containing body, showing many small circular membrane structures (arrowheads) surrounded by an outer membrane (arrow). **D** Three *Wolbachia* (arrows) in the vicinity of WSP*-*1 positive vesicles (arrowheads). **E** *Wolbachia* with gold particles labeling the outer membrane (arrow) and vesicles (arrowhead). **F** Gold particles labeling a large *Wolbachia*-derived vesicle and adjacent membrane fragments (arrowheads). **G** Higher power view of one *Wolbachia* indicating labeling of the inner *Wolbachia* membrane (arrow) and outer membrane vesicles (arrowhead). Scale bar corresponds to 100 nm.

Some morphological forms of *Wolbachia* in the lateral chord and in early embryos of *B. malayi* synthesize and release membrane vesicles (Table 1). Large vesicles with diameters of 80–160 nm are released by electron dense coccoid *Wolbachia* in morula stage embryos in the uterus of 12 week old females (Fig. 3). These vesicles have a low electron density and often have two membranes when they are released into the cytoplasm (Fig. 3D, E). Vesicles with a single bilayer membrane were frequently observed within an outer vacuole membrane together with *Wolbachia* (Fig. 3B, C, F). This suggests that these vesicles are produced by the bacteria, especially in areas devoid of organelles such as the Golgi apparatus. In addition to single vesicles we saw polymorphic structures, where a number of smaller vesicles are enclosed by a large outer membrane (Fig. 4A–C, Figs. S1, S2). While the formation of membrane vesicles has not been specifically studied in filarial *Wolbachia*, it is well known that Gram-negative bacteria release outer membrane vesicles. Vesicle formation is often associated with detoxification and acquisition of nutrients [33] or with virulence in pathogenic bacteria [34], [35]. Although isolation of membrane vesicles is challenging, vesicles of the intracellular pathogen *Chlamydia trachomatis* have been isolated from host cells [36]. However, different subpopulations of vesicles in the vicinity of *C. trachomatis* have been described and some of them are derived from host cells. So far the detailed content of vesicles is known only for very few bacteria species [37].

### Immunogold Labeling of *Wolbachia* and *Wolbachia*-derived Vesicles

Although the localization of many vesicles suggested that *Wolbachia* are synthesizing and releasing the vesicles, it is possible that some of these vesicles in close proximity to *Wolbachia* were formed by host organelles and taken up by the endobacteria. Therefore, we used a monoclonal antibody (mab) directed against *Wolbachia* surface protein 1 (WSP-1) to assess the origin of these vesicles (Fig. 4). As a technical control an antibody against double stranded (ds) DNA was used that showed specific labeling of dsDNA in host’s nuclei and in *Wolbachia* cells while resulting in very little background (Fig. S3) The experiments using mab WSP-1 showed that *Wolbachia* membranes, including a large number of vesicles in host cell cytoplasm in the vicinity of *Wolbachia*, were labeled by mab WSP-1. The surface protein is localized at the outer, vacuole membrane which had previously thought to be formed by the host [1]. However, it is not clear how *Wolbachia* transfer WSP-1 into a host-derived membrane. Gold particles were seen only in association with membranes, but membranes of vesicles present in tissues devoid of *Wolbachia* were not labeled. Immunostaining affected the integrity of the membranes slightly, and the morphological preservation was not as good as in the regular TEM. However, no ‘free’ or non-membrane bound WSP-1 was detected, and this result differs from results recently reported by other authors [17]. Even membranes of multivesicular structures in the vicinity of *Wolbachia* were WSP-1 positive (Fig. 4A–D). A recent ultrastructural study using chemically fixed specimens suggested that WSP-1 (wBm0432) is co-localized with aldolase and interacts with the host’s glycolytic pathway [26]. A bilayer membrane expected to be present in traditional bound vacuoles was not detected and the authors hypothesized that their specimens were not preserved well enough to demonstrate membrane binding of the antibody. Our results indicate that vesicle formation by *Wolbachia* is common and that WSP-1 is membrane-bound and localized to membranes forming the *Wolbachia* vacuole and *Wolbachia* derived vesicles.

### Relation of *Wolbachia* to Organelles of the Host’s Secretory Pathway

**Figure 5:**
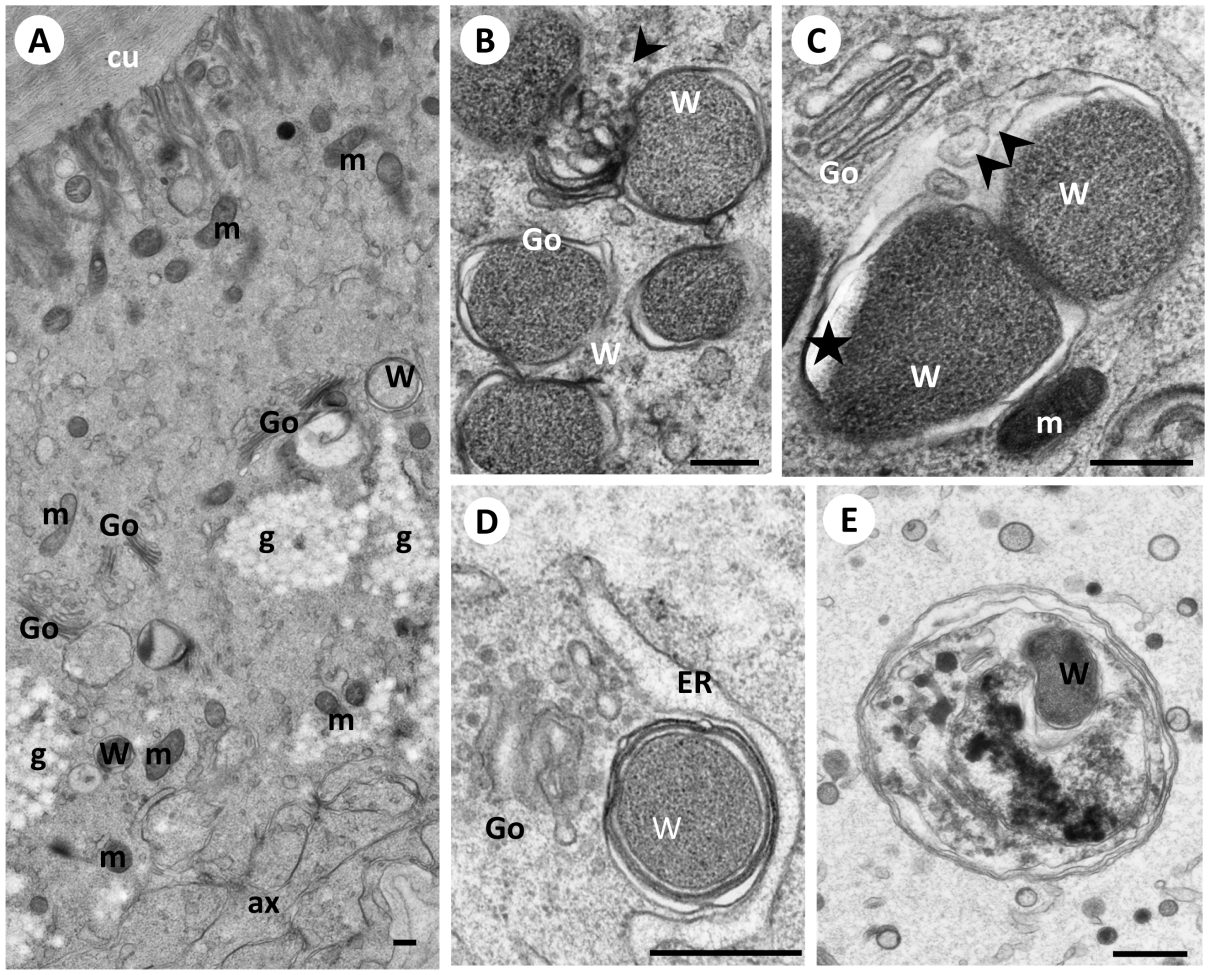
*Wolbachia* organelles in the lateral chord of 5 weeks (D,E) and 12 weeks (A,B,C) old female *B. malayi*. **Panel A** Overview of the hypodermal chord showing few *Wolbachia* in the central region sometimes in the vicinity of the Golgi apparatus. **B** Two *Wolbachia* in close contact to Golgi between them an accumulation of vesicles (arrowhead). **C** In close proximity to Golgi potentially dividing *Wolbachia* with vesicles (arrowheads within the endosomal membrane. Asterisk indicates an artifact. **D** *Wolbachia* surrounded by multiple membranes in close proximity to the endoplasmic reticulum and the Golgi apparatus. **E** *Wolbachia* inside a phagolysosome. Cu, cuticle; m, mitochondrion; Go, Golgi; g, glycogen; W, *Wolbachia*; ax, axons;ER, endoplasmic reticulum. Scale bar corresponds to 500 nm.

Pathogen-containing intracellular compartments sometimes interact with secretory pathways of the host cell [38]. Therefore, we studied the association of *Wolbachia* with host organelles such as the endoplasmic reticulum and Golgi apparatus. HPF/FS resulted in excellent preservation of these subcellular structures. Single or small clusters of *Wolbachia* within the lateral chord were frequently detected in the vicinity of the Golgi apparatus, the endoplasmic reticulum, and inside or outside of phagolysosomes (Fig. 5, Figs. S1, S2). Single (Fig. 5B) or dividing (Fig. 5C) *Wolbachia* and vesicles were observed on both the cis and the trans sides of the Golgi apparatus. For comparison, it has been reported that *Chlamydia* disrupts the Golgi apparatus to ensure reproduction, while some insect *Wolbachia* reside within Golgi-related vesicles [39], [40]. A *Drosophila Wolbachia* strain has been reported to be associated with the endoplasmic reticulum [41]. We often observed small numbers of *Wolbachia* near nucleus-associated endoplasmic reticulum in the lateral chord (Fig. 5D). However, large clusters of *Wolbachia*, which are often seen in the lateral chord of L4 and young adult worms during the exponential growth of the endobacteria population, were rarely seen in direct contact with the endoplasmic reticulum or the Golgi apparatus. Based on these observations, it is possible that *Wolbachia* use the secretory pathway of the host cell including vesicle trafficking and membrane fusion for intracellular and syncytial motility.

### *Wolbachia* in the Lateral Chord and in the Pseudocoelom

**Figure 6:**
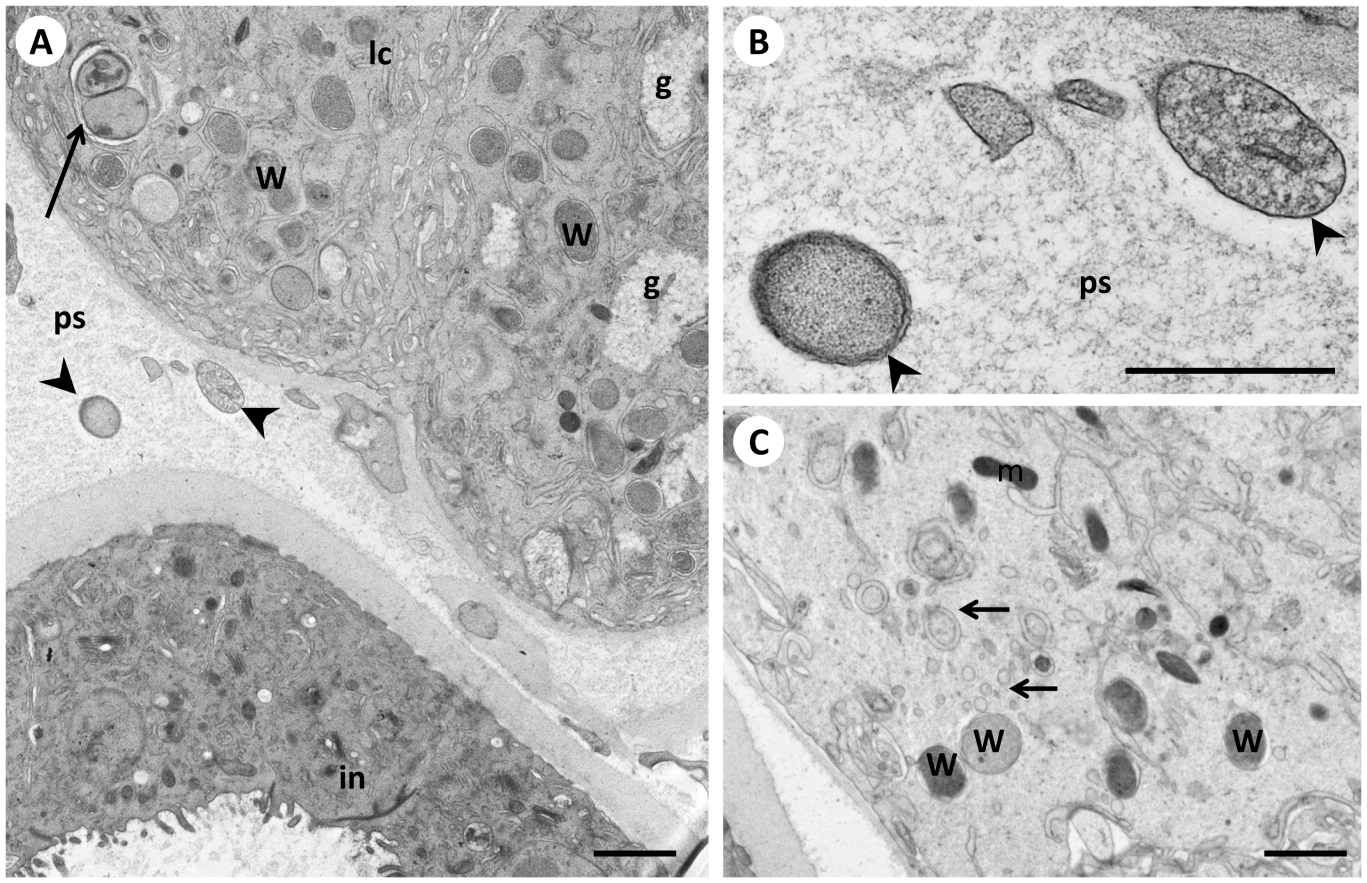
*Wolbachia* in the lateral chord and the pseudocoelomic cavity of a 6 weeks old female *B. malayi*. **Panel A** Overview of the lateral chord and one uterus branch at the distal part of the worm showing numerous *Wolbachia* in the lateral chord and few extracellular *Wolbachia* (arrowheads) in the pseudocoelomatic cavity. Note the two large dividing endobacteria (arrow). **B** Close-up of the two extracellular *Wolbachia* from panel A showing only one thick cell envelope (arrowheads). **C** Another part of the lateral chord showing *Wolbachia* and numerous single and double membrane vesicles (arrows). W, *Wolbachia*; g, glycogen; ps, pseudocoelom; m, mitochondrion, lc, lateral, chord; in, intestine. Scale bar corresponds to 500 nm.

Large numbers of *Wolbachia* were found in the lateral chord in the posterior end of the worm (near the ovaries and the uterus) in young, immature female *B. malayi* (Fig. 6). Extracellular *Wolbachia* were sometimes present in the pseudocoelomic cavity (Fig. 6A). This observation is consistent with those recently reported (compare to Fig. 8 in [12]). HPF/FS fixation showed that these extracellular *Wolbachia* have a thick cell envelope comprised of two membranes (Fig. 6B). The utrastructural images did not reveal a mode of motility for these extracellular *Wolbachia*; it is possible that passive motility based on changes in pressure and flow of fluid in the pseudocoelomic cavity enable bacteria to invade the reproductive system of the worm host. It is also possible that these extracellular *Wolbachia* forms induce endocytosis by host cells in a manner similar to elementary bodies of *Chlamydia*.

**Figure 7:**
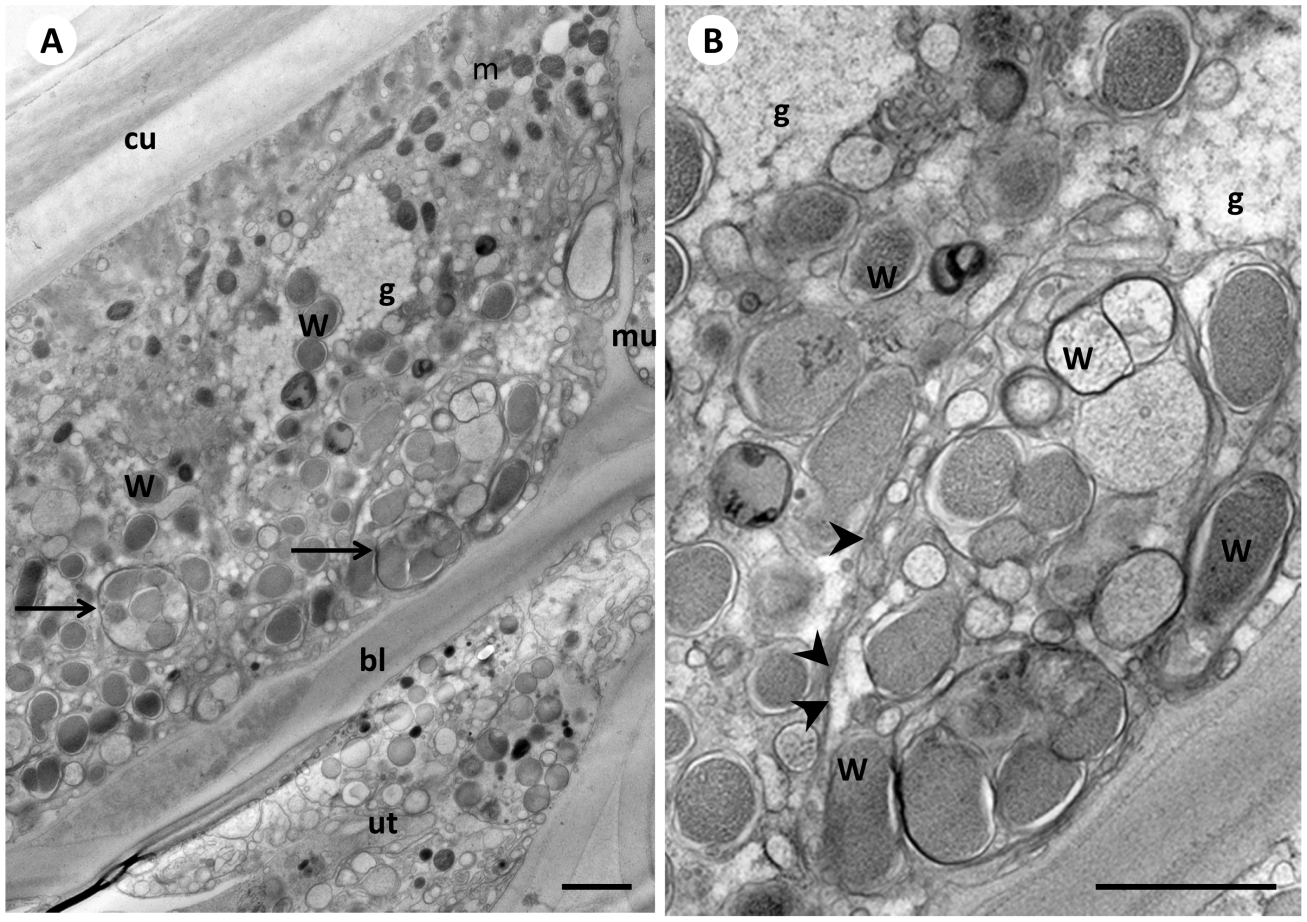
*Wolbachia* in the distal part of the lateral chord of a female 12 weeks old *B. malayi*. **Panel A** Sagittal section showing numerous single and clustered *Wolbachia* (arrows). While *Wolbachia* were abundant in the posterior quarter of the female’s lateral chord (paralleling the ovaries, oviducts and the uteri containing early stage embryos) only a few single endobacteria were seen in the lateral chord at the anterior end of the worm. **B** Higher power view of panel A showing small vesicles, single *Wolbachia*, and small ‘inclusion-like’ *Wolbachia* clusters that appear to be enclosed by a common membrane (arrowheads). W, *Wolbachia*; Cu, cuticle, m, mitochondrion; mu, muscles; bl, basal lamina; ut, uterus; g, glycogen. Scale bar corresponds to 1 µm.

*Wolbachia* have their highest density and greatest morphological diversity in the lateral chords of female L4, in immature adult females, and in mature females adjacent to the distal portion of the reproductive organs, especially ovary, oviduct and seminal receptacle. In some parts of the lateral chord, the tissue is highly vacuolized and consists almost exclusively of *Wolbachia* either as single cells or as clusters (Fig.7). These dense accumulations of *Wolbachia* are often associated with high densities of glycogen granules. Physical proximity of *Wolbachia* and glycogen suggest a functional association. Transcriptomic and molecular biological studies of filarial *Wolbachia* have suggested a mitochondrion-like function [26], [28], [42]. However, direct consumption of glucose or glycogen by *Wolbachia* seems unlikely, because its genome lacks genes coding for two enzymes required for glycolysis [28].

## Conclusions

HPF/FS fixation dramatically improved tissue preservation for enhanced ultrastructural examination of *Wolbachia* and filarial nematodes. Our study provides more detailed information regarding the pleomorphy of *Wolbachia* in different tissues, their association with vesicles, and their movement within cells and across tissues. Membrane and membrane vesicles appear to be critically important morphological structures involved in the *Wolbachia*/filarial nematode interactions. Vesicles may shuttle metabolic products between bacteria and worm tissues, this could help explain the interdependence of and *B. malayi*.

## Methods

### Parasite Material

The protocol to maintain *B. malayi* in Mongolian gerbils for morphological studies was approved by the Animal Studies Committee at Washington University School of Medicine. Male gerbils were infected i.p. with 200 *B. malayi* infective larvae and sacrificed 2, 3, 4, 5, 6, 8 and 12 weeks post infection. Parasites were obtained in intraperitoneal lavage using PBS cooled to 4°C and older stages were separated by sex. After external examination worms were immediately submitted for HPF/FS. For each time point at least 5 larvae or 3 female worms were examined.

### Standard Processing for TEM

For conventional ultrastructural analysis of chemically fixed specimens, live worms were fixed in 2% paraformaldehyde/2.5% glutaraldehyde (Polysciences Inc., Warrington, PA) in 100 mM phosphate buffer, pH 7.2 for 1 hr at room temperature. Samples were washed in phosphate buffer and postfixed in 1% osmium tetroxide (Polysciences Inc.) for 1 hr. Samples were then rinsed extensively in distilled water prior to en bloc staining with 1% aqueous uranyl acetate (Ted Pella Inc., Redding, CA) for 1 hr. Following several rinses in distilled water, samples were dehydrated in a graded series of ethanol and embedded in Eponate 12 resin (Ted Pella Inc.). Sections of 95 nm were cut with a Leica Ultracut UCT ultramicrotome (Leica Microsystems Inc., Bannockburn, IL), stained with uranyl acetate and lead citrate, and viewed on a JEOL 1200 EX transmission electron microscope (JEOL USA Inc., Peabody, MA) equipped with an AMT 8 megapixel digital camera (Advanced Microscopy Techniques, Woburn, MA). Figure plates were assembled using Adobe Photoshop Elements 8.0. Images were not computationally enhanced except for adjustments for brightness and contrast.

### High-pressure Freezing and Freeze Substitution (HPF/FS)

Live worms were placed in specimen planchettes containing 20% bovine serum albumin as a cryoprotectant. Planchettes were then high-pressure frozen in a Leica EM PACT2 high-pressure freezer (Leica Microsystems) at −180°C, 2,100 bar and maintained under liquid nitrogen. For optimal ultrastructural analysis, samples were transferred to freeze substitution medium (acetone containing 2% osmium tetroxide, 0.1% uranyl acetate, and 5% distilled water) under liquid nitrogen and placed in the Leica AFS, automatic freeze substitution system (Leica Microsystems) precooled to −130°C. For freeze substitution, the samples were brought to −90°C over 1 hr, remaining at −90°C for 10 hr, and subsequently warmed to −20°C over a period of 18 hr. Samples were placed at 4°C for 30 min prior to washing with anhydrous acetone at room temperature. Samples were infiltrated and embedded in Eponate 12 resin, and sectioned and stained as described above.

### Immunogold TEM

For immunolabeling of high-pressure frozen samples, the freeze substitution medium consisted of acetone containing 0.2% glutaraldehyde, 0.1% uranyl acetate, and 5% distilled water. Following freeze substitution in the AFS unit as described above, samples were infiltrated in LR Gold resin (Ted Pella) at −20°C, polymerized under UV light, and subsequently sectioned and stained. A mab against WSP-1 (Wbm0432) was used. The antibody was kindly provided by Patrick Lammie (CDC, Atlanta) and extensively evaluated by immunohistochemistry previously [12]. A monoclonal antibody directed against ds DNA (clone AC-3-10, Novus Biologicals, CO) was diluted 1∶20 and used as a positive control according to the instructions of the manufacturer.

## Supporting Information

Figure S1 **Ultrastructural evidence for** ***Wolbachia*** **producing vesicles in the lateral chord of an 8 weeks old** ***B. malayi*** **female. Panel A** Pleomorphic *Wolbachia* close to a large nucleus. Note the large endoplasmic reticulum with and without ribosomes. Various vesicles (arrowheads) are within or attached to the vacuole membrane. **B** Higher power view of part of panel **A** showing also Golgi cisternae proximal to *Wolbachia* and endoplasmic reticulum. **C** Another part of panel **A** showing *Wolbachia* near single (arrowhead) and multiple (double arrowheads) vesicles. **D** Magnification of a portion of panel **A** demonstrating a *Wolbachia* enclosed by multiple membranes with numerous small vesicles (arrowhead) surrounded by the same outer membrane. This structure may be similar to those in panel **C**, but from a different perspective. W, *Wolbachia*; nu, nucleus; sER, smooth endoplasmic reticulum; rER, rough endoplasmic reticulum; Go, Golgi; Scale bar corresponds in **A–C** to 500 nm and in **D** to 200 nm.
(TIF)

Figure S2 **Different forms of vesicles associated with** ***Wolbachia*** **in the lateral chord of young adult** ***B. malayi*** **females. Panel A** Two small vesicles (arrowheads) surrounded by the same vacuole membrane as a large *Wolbachia*. Note that the endobacteria is next to the trans side of the Golgi. **B** *Wolbachia* with an attached multi vesicular structure (arrowhead). **C** Higher power view of B showing the attachment region (arrowhead). **D** Some *Wolbachia* in the vicinity of another multi vesicular structure (arrowheads). **E** A similar view to that shown in A, that indicates an interaction of *Wolbachia* and vesicles (arrowheads) with the Golgi. W, *Wolbachia*; m, mitochondrion; Go, Golgi; nu, nucleus. Scale bar corresponds in **A–D** to 100 nm and in **E** to 500 nm.
(TIF)

Figure S3 **Immunogold-labeling of double stranded DNA in** ***B. malayi*** **and** ***Wolbachia*** **cells using mab against dsDNA (clone AC-30-10) as a technical control experiment. Panel A** Nucleus of a morula stage embryo within the uterus of a female *B. malayi* showing electron-dense chromatin labeled by mab dsDNA (arrows). **B** Coss-section of an early spermatozoa of a 8-week old male *B. malayi* showing electron-dense, labeled chromosomes (arrow). **C** Loose cluster of *Wolbachia* in the lateral chord of an adult female *B. malayi*. Note the gold particles (arrow) in the endobacteria indicating the presence of dsDNA and the absence of any labeling in the surrounding tissue. **D** Close-up of another region similar to C showing the highly specific labeling. Scale bar corresponds to 100 nm.
(TIF)
