# Supplementary figures and images for: High Pressure Freezing/Freeze Substitution Fixation Improves the Ultrastructural Assessment of Wolbachia Endosymbiont – Filarial Nematode Host Interaction
Source: PLoS One. 2014 Jan 17;9(1):e86383. doi: 10.1371/journal.pone.0086383 (PMC3895037; doi:10.1371/journal.pone.0086383)

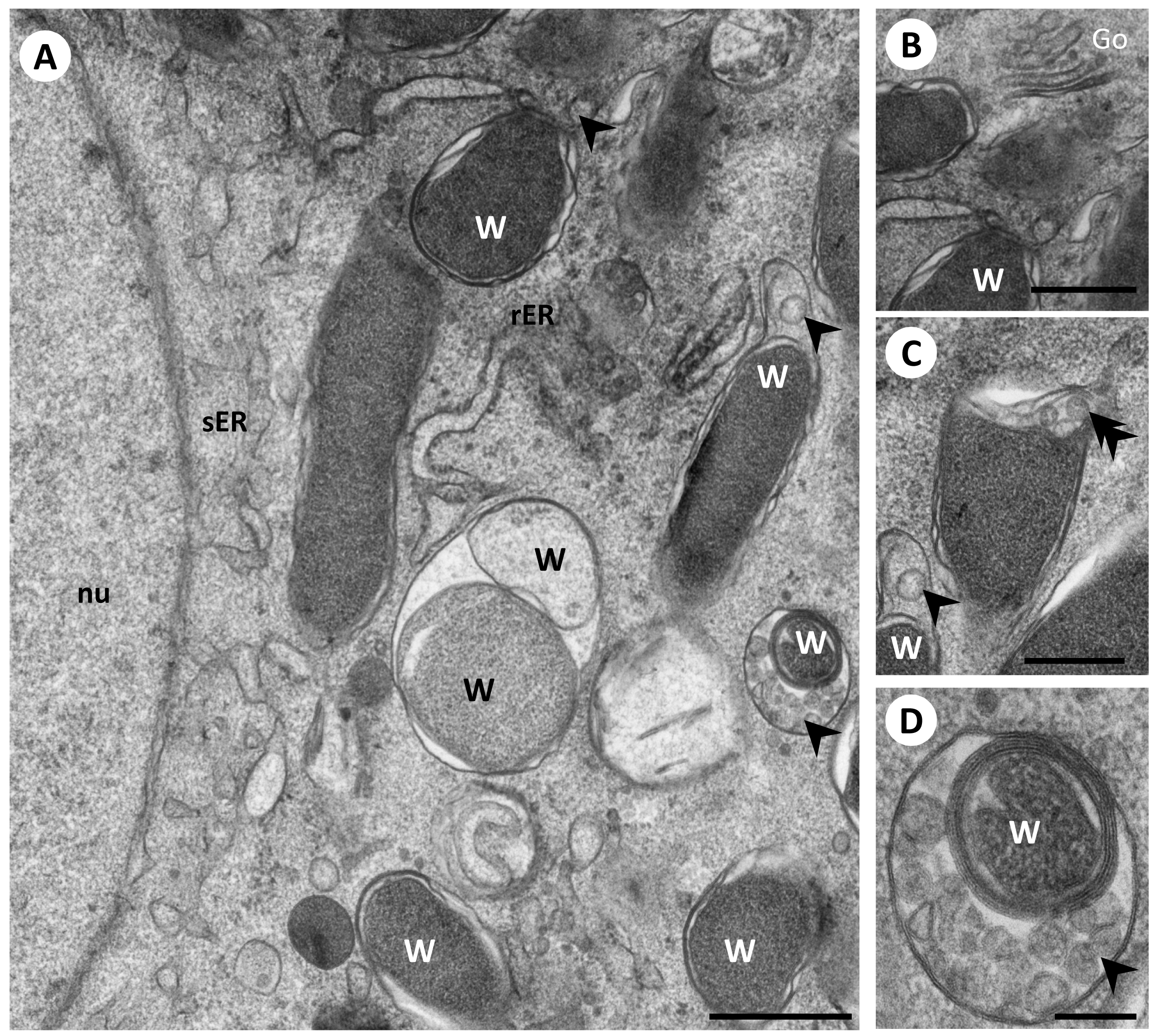

Supplement: Figure S1 — Ultrastructural evidence for Wolbachia producing vesicles in the lateral chord of an 8 weeks old B. malayi female. Panel A Pleomorphic Wolbachia close to a large nucleus. Note the large endoplasmic reticulum with and without ribosomes. Various vesicles (arrowheads) are within or attached to the vacuole membrane. B Higher power view of part of panel A showing also Golgi cisternae proximal to Wolbachia and endoplasmic reticulum. C Another part of panel A showing Wolbachia near single (arrowhead) and multiple (double arrowheads) vesicles. D Magnification of a portion of panel A demonstrating a Wolbachia enclosed by multiple membranes with numerous small vesicles (arrowhead) surrounded by the same outer membrane. This structure may be similar to those in panel C, but from a different perspective. W, Wolbachia; nu, nucleus; sER, smooth endoplasmic reticulum; rER, rough endoplasmic reticulum; Go, Golgi; Scale bar corresponds in A–C to 500 nm and in D to 200 nm. (TIF) [file pone.0086383.s001.tif]

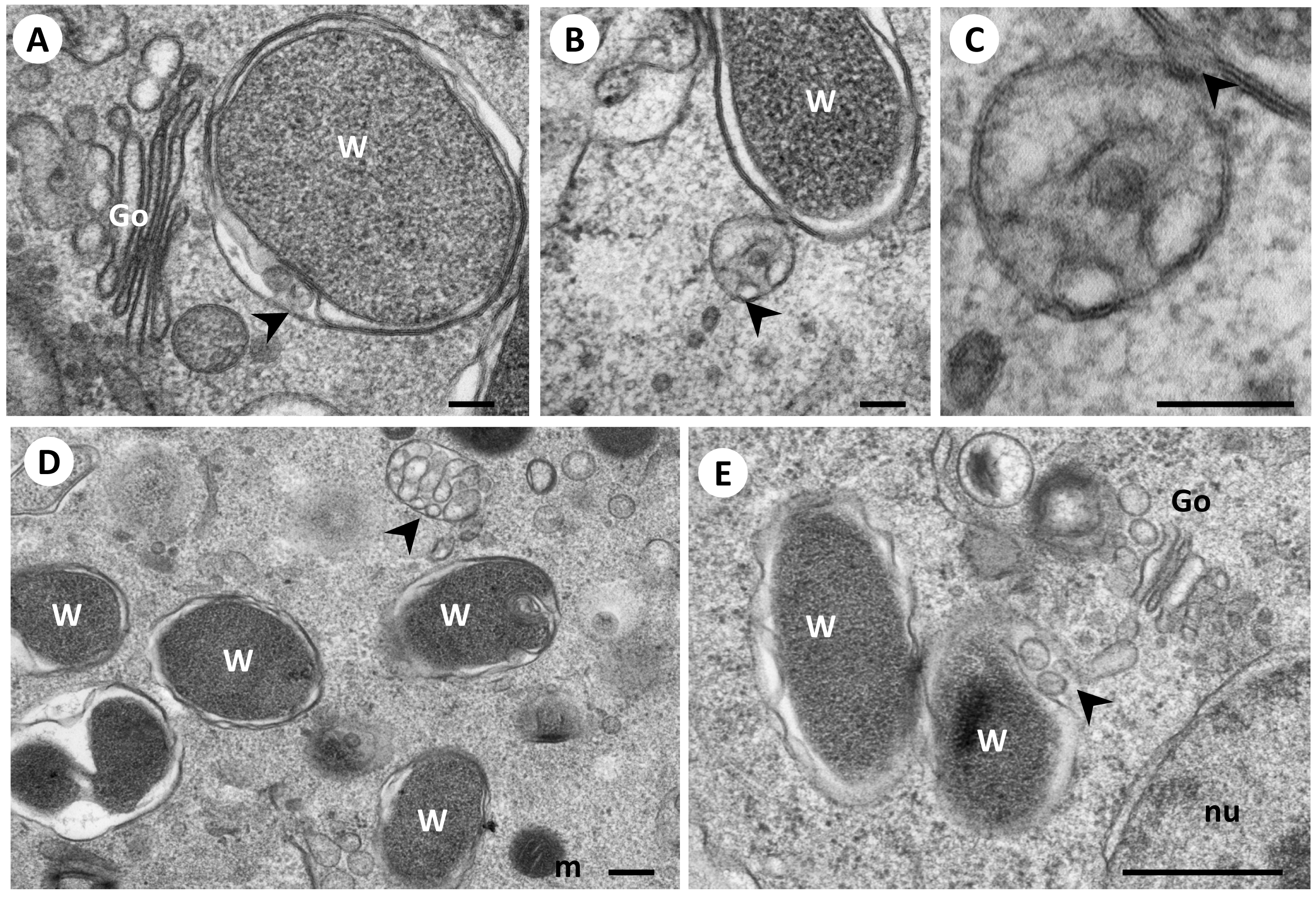

Supplement: Figure S2 — Different forms of vesicles associated with Wolbachia in the lateral chord of young adult B. malayi females. Panel A Two small vesicles (arrowheads) surrounded by the same vacuole membrane as a large Wolbachia. Note that the endobacteria is next to the trans side of the Golgi. B Wolbachia with an attached multi vesicular structure (arrowhead). C Higher power view of B showing the attachment region (arrowhead). D Some Wolbachia in the vicinity of another multi vesicular structure (arrowheads). E A similar view to that shown in A, that indicates an interaction of Wolbachia and vesicles (arrowheads) with the Golgi. W, Wolbachia; m, mitochondrion; Go, Golgi; nu, nucleus. Scale bar corresponds in A–D to 100 nm and in E to 500 nm. (TIF) [file pone.0086383.s002.tif]

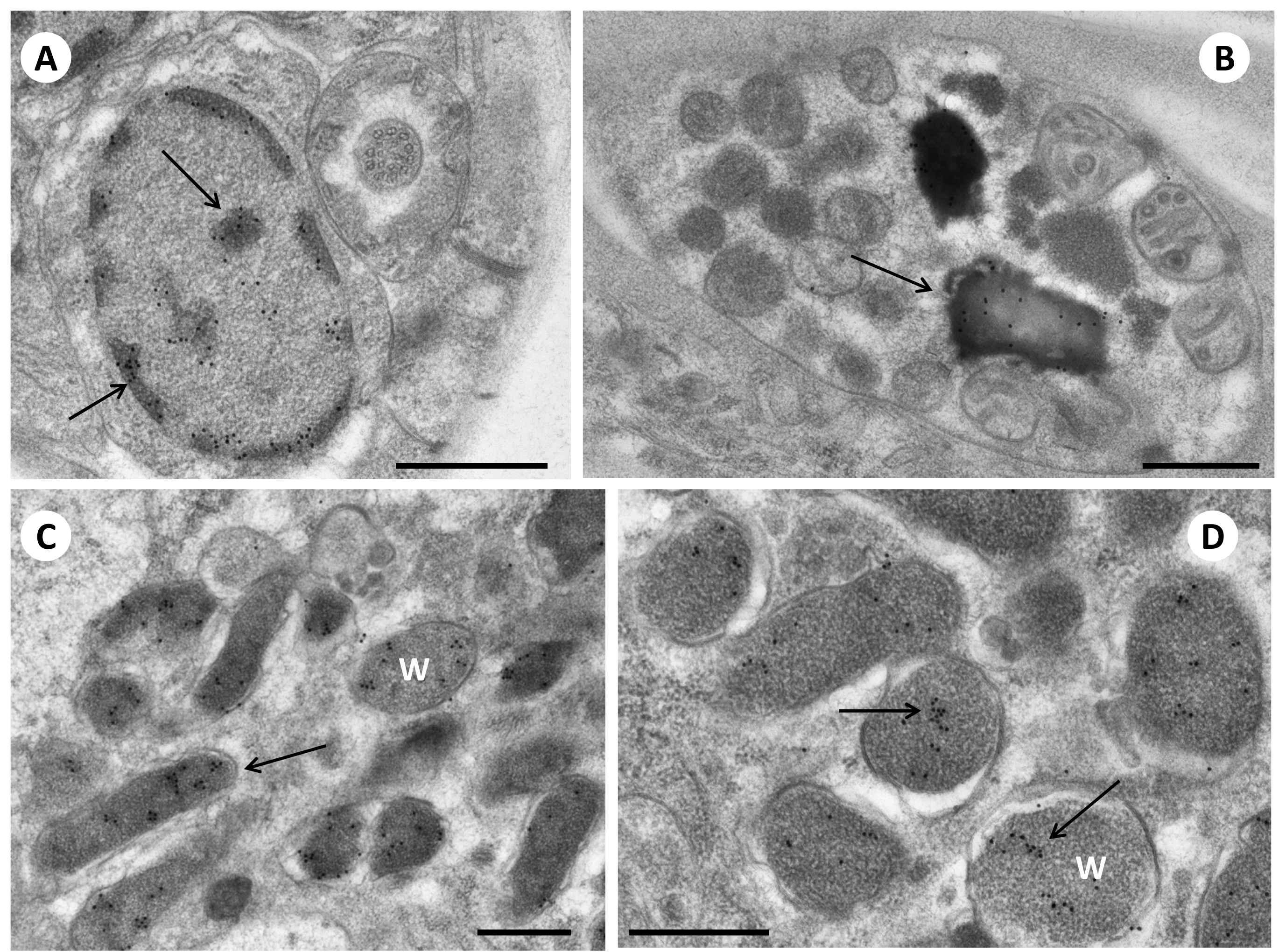

Supplement: Figure S3 — Immunogold-labeling of double stranded DNA in B. malayi and Wolbachia cells using mab against dsDNA (clone AC-30-10) as a technical control experiment. Panel A Nucleus of a morula stage embryo within the uterus of a female B. malayi showing electron-dense chromatin labeled by mab dsDNA (arrows). B Coss-section of an early spermatozoa of a 8-week old male B. malayi showing electron-dense, labeled chromosomes (arrow). C Loose cluster of Wolbachia in the lateral chord of an adult female B. malayi. Note the gold particles (arrow) in the endobacteria indicating the presence of dsDNA and the absence of any labeling in the surrounding tissue. D Close-up of another region similar to C showing the highly specific labeling. Scale bar corresponds to 100 nm. (TIF) [file pone.0086383.s003.tif]
